# Deep Proteome Profiling with Reduced Carryover Using Superficially Porous Microfabricated nanoLC Columns

**DOI:** 10.1021/acs.analchem.2c01196

**Published:** 2022-11-10

**Authors:** Karel Stejskal, Op de Beeck Jeff, Manuel Matzinger, Gerhard Dürnberger, Alexander Boychenko, Paul Jacobs, Karl Mechtler

**Affiliations:** † IMP—Institute of Molecular Pathology, Campus-Vienna-Biocenter 1, A-1030 Vienna, Austria; ‡ IMBA—Institute of Molecular Biotechnology of the Austrian Academy of Sciences, Dr. Bohr Gasse 3, A-1030 Vienna, Austria; § Gregor Mendel Institute of Molecular Plant Biology of the Austrian Academy of Sciences, Dr. Bohr Gasse 3, A-1030 Vienna, Austria; ∥ Thermo Fisher Scientific, Technologiepark-Zwijnaarde 82, B-9052 Gent, Belgium; ⊥ Thermo Fisher Scientific, Dornierstrasse 4, 82110 Germering, Germany

## Abstract

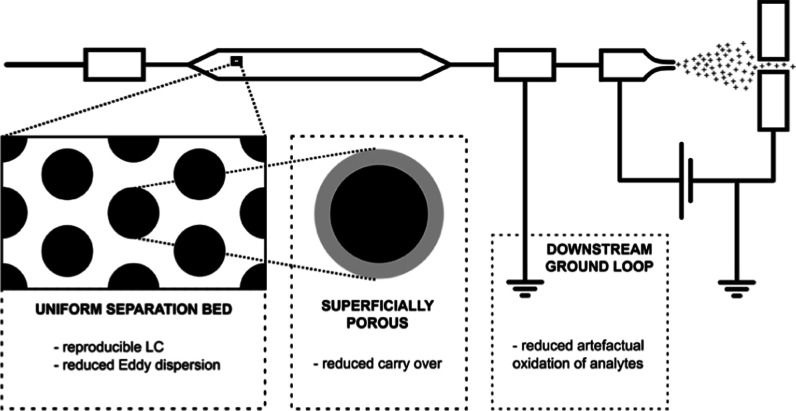

In the field of liquid chromatography–mass spectrometry (LC–MS)-based proteomics, increases in the sampling depth and proteome coverage have mainly been accomplished by rapid advances in mass spectrometer technology. The comprehensiveness and quality of the data that can be generated do, however, also depend on the performance provided by nano-liquid chromatography (nanoLC) separations. Proper selection of reversed-phase separation columns can be important to provide the MS instrument with peptides at the highest possible concentration and separated at the highest possible resolution. In the current contribution, we evaluate the use of the prototype generation 2 μPAC nanoLC columns, which use C18-functionalized superficially porous micropillars as a stationary phase. When compared to traditionally used fully porous silica stationary phases, more precursors could be characterized when performing single shot data-dependent LC–MS/MS analyses of a human cell line tryptic digest. Up to 30% more protein groups and 60% more unique peptides were identified for short gradients (10 min) and limited sample amounts (10–100 ng of cell lysate digest). With LC–MS gradient times of 10, 60, 120, and 180 min, respectively, we identified 2252, 6513, 7382, and 8174 protein groups with 25, 500, 1000, and 2000 ng of the sample loaded on the column. Reduction of sample carryover to the next run (up to 2 to 3%) and decreased levels of methionine oxidation (up to 3-fold) were identified as additional figures of merit. When analyzing a disuccinimidyl dibutyric urea-crosslinked synthetic library, 29 to 59 more unique crosslinked peptides could be identified at an experimentally validated false discovery rate of 1–2%.

## Introduction

Even though the practice of liquid chromatography–mass spectrometry (LC–MS)-based bottom-up proteomics has remained relatively unaltered over the past decade, researchers are progressively closing the gap between experimentally identified and theoretically expected proteoforms present in complex cell lysates.^1−3^ Key aspects driving this progress are the continuous evolution of MS/MS instruments, the coming of age of additional ion mobility separation techniques, and the combination with LC separation that delivers maximal resolving power and throughput.^1,4,5^ Even though MS/MS instruments have evolved to a point where acquisition rates up to 133 Hz can be reached,^6,7^ these developments have struggled to materialize similar leaps in proteome coverage depth, such as those obtained by publications of Thakur et al., Hebert et al., and Scheltema et al. in 2011 and 2014.^8−10^ As postulated several years ago by Shishkova et al.,^11^ chromatographic separation performance is the key, but it perhaps is an underappreciated bottleneck limiting the speed and depth of single-shot proteomic analyses. Improvements in the chromatographic resolution have historically been achieved by increasing the column length or by decreasing silica particle diameters.^12−14^ However, reducing particle diameters and extending the column length have a synergistic effect on operating pressures.^15,16^ Consequently, current state-of-the art nanoLC columns often require ultra-high pressure liquid chromatography (UHPLC) instruments that can accurately deliver nanoliter per minute flow rates at operating pressures of up to 1500 bar.

To cope with these pressure requirements, alternative formats, such as monolithic columns, have been introduced, albeit with limited adoption in the field of proteomics.^17−19^ Alternatively, microfabricated pillar array columns (μPAC) have been proposed as a new promising technology that can redefine the boundaries of LC performance.^20^ Using micromachining techniques rather than slurry packing, both the chromatographic performance and column permeability can be controlled by design. We describe the use of a new generation of pillar array columns whose design specifications have been tightened in search of increased separation performance. Schematic drawings of the “building” blocks or unit cells used to design different “generations” of pillar array columns are shown in Figure S1. Analogous to observations in packed bed columns, reduction of the pillar and inter pillar dimensions by a factor of 2 results in a net gain in separation resolution with a factor of 1.4 at the cost of increased operating pressure.^21^ In contrast to the experiments we conducted for limited sample amounts in 2021,^22^ the work we report on in the current contribution uses a superficially porous rather than a nonporous version of the generation 2 pillar array column. By using electrochemical anodization, the outer shell of the cylindrical pillars is rendered mesoporous with pore sizes in the range of 100–300 Å. This increases the available interaction surface by a factor of approximately 30, making this format more compatible with conventional sample loads.

To investigate potential benefits of the μPAC column for nanoLC–MS applications, we report on an extensive benchmarking series, where we coupled this column to the latest generation of tribrid MS systems, a field asymmetric waveform ion mobility spectrometry (FAIMS) pro interface, and a next-generation low-flow UHPLC system (Vanquish Neo UHPLC). Such experiments are commonly performed with highly validated mammalian protein digest standards to provide unbiased data on instrument performance. Results do, however, often differ from what can be achieved with biologically relevant samples and fail to provide information on day-to-day robustness and throughput. The current study aims to address these matters by providing additional data on performance over time, column-related sample carryover, and validation of results by implementing the workflow for the analysis of a synthetic library of cross-linked peptides.^23^

## Experimental Section

### Sample Preparation

Pierce HeLa Protein Digest Standard (Thermo Fisher Scientific) was used for MS parameter optimization as well as for final column benchmarking measurements. 20 μg of peptide pellets were dissolved in LC/MS grade water with 0.1% (v/v) trifluoroacetyl (TFA) and diluted to the required peptide concentration in autosampler vials (Fisherbrand 9 mm Short Thread TPX Vial with integrated Glass Micro-Insert; Cat. no. 11515924). All liquid handling was done as fast as possible without unnecessary time gaps, with the aim to minimize sample losses on plastics and glass surfaces.

For the cross-linking experiment, synthetic peptides generated by Beveridge and coworkers were cross-linked using disuccinimidyl dibutyric urea (DSBU), as described in their paper.^23^ The final cross-linked peptide mix was merged either with an equal amount of tryptic HeLa peptides (Pierce HeLa Protein Digest Standard dissolved in 0.1% TFA) to obtain a 1:1 spiked system or with 5 times the amount of tryptic HeLa peptides to obtain a 1:5 spiked system. A total amount of 1 μg (either using the cross-linked peptide mix only or total peptide after spiking) of the peptide was used for each LC–MS/MS analysis.

### Liquid Chromatography–Mass Spectrometry Analysis

Peptide samples were analyzed using a Vanquish Neo UHPLC instrument in the nano/cap mode and configured for direct injection onto the column. The Orbitrap Eclipse Tribrid mass spectrometer was equipped with the FAIMS Pro interface (Thermo Fisher Scientific). Peptides were separated with either the new generation prototype 50 cm pillar array column (Thermo Fisher) or with a 25 cm long packed bed column with an integrated tip.

The 50 cm μPAC was placed in a Butterfly heater (PST-BPH-20, Phoenix S&T) and operated at 50 °C. The column was connected to an EASY-Spray bullet emitter (10 μm ID, ES993; Thermo Fisher Scientific) with a custom-made fused silica capillary (20 μm ID × 360 μm OD, length 10 cm, Polymicro) with 1/16″ ZDV fittings and a precision-cut PEEK sleeve on the ESI source-facing side. An electrospray voltage of 2.4 kV was applied at the integrated liquid junction of the EASY-Spray emitter. To prevent electric current from affecting the upstream separation column, a 50 μm internal bore stainless steel reducing union (VICI; C360RU.5S62) was electrically connected to the grounding pin at the pump module (Figure S2).

The packed bed analytical column (25 cm × 75 μm ID, 1.6 μm C18; AUR2-25075C18A; IonOpticks) was installed in a Sonation column oven (PRSO-V2; Sonation) and operated at 50 °C. The Sonation column oven was mounted on a NanoFlex ion source (Thermo Fisher Scientific). An electrospray voltage of 2.4 kV was applied at the nanoZero fitting via the high voltage cable (HVCABLE01; IonOpticks) (Figure S2).

Peptides were separated with stepped linear solvent gradients; all were performed at a flow rate of 200 nL/min (except the flow rate experiment) with various durations of 10, 60, 120, and 180 min. The organic modifier content (acetonitrile acidified with 0.1% v/v formic acid) was first increased from 0.8 to 18% in 7.5, 45, 90, and 135 min; then increased from 18 to 32% in 2.5, 15, 30, and 15 min; and finally ramped from 32 to 76% in 5 min. The mobile phase composition was kept at a high organic phase (76% acetonitrile acidified with 0.1% v/v formic acid) for 5 min to wash the column. Column re-equilibration was performed at a low organic phase (0.8% acetonitrile acidified with 0.1% v/v formic acid) with 2 column volumes.

### MS Acquisition

The mass spectrometer was operated in the data-dependent mode using a full scan with an *m*/*z* range of 375–1500, an orbitrap resolution of 120.000, a target value 250%, and the maximum injection time set to auto. Compensation voltages of −45, −55, and −75 V or −45, −55, −65, and −75 V were combined in a single run with total cycle times of 3 or 4 s, respectively. The intensity threshold for precursor was set to 5 × 10^4^. Dynamic exclusion duration was based on the length of the LC gradient set up for 10 min to 20 s, for 60 min to 25 s, for 120 min to 40 s, and for 180 min to 60 s.

MS/MS spectra were acquired in the ion trap analyzer and fragmented by stepped higher-energy collisional dissociation using a normalized collision energy of 30%. Precursors were isolated in a window of 1.0 Da. The linear ion trap acquired spectra in the turbo mode and in the range of 200–1400 *m*/*z*. The normalized AGC target was set to 300%, and the maximum injection time was 12.5 ms for 10 and 60 min long gradient methods and 15 ms for 120 and 180 min long gradient methods.

### Data Analysis

MS/MS spectra from raw data were imported to Proteome Discoverer (PD) (version 2.5.0.400, Thermo Scientific). First, spectra were recalibrated in the PD node “Spectrum Files RC” using the human SwissProt database (*Homo sapiens*; release 2020_12; 20,541 sequences and 11,395,748 residues) and a database of common contaminants (375 sequences and 144,816 residues). Recalibration was performed for fully tryptic peptides by applying an initial precursor mass tolerance of 20 ppm and a fragment mass tolerance of 0.5 Da. Carbamidomethylation of cysteine was set as a fixed modification in the recalibration step. A database search on individual raw files was performed using MS Amanda^24^ (version 2.5.0.16129) and the FASTA databases already described above at recalibration. Trypsin was specified as a proteolytic enzyme, cleaving after lysine (K) and arginine (R) except when followed by proline (P), and up to one missed cleavage was considered. Mass tolerance was limited to 7 ppm at the precursor level and 0.3 Da at the fragment level. Carbamidomethylation of cysteine (C) was set as a fixed modification, and oxidation of methionine (M), as well as acetylation and the loss of methionine at the protein N-terminus, were set as a variable modification. Identified spectra were rescored using a Percolator,^25^ as implemented in PD, and filtered for a 1% false discovery rate (FDR) at the peptide spectrum match and peptide levels. Abundance of identified peptides was determined by label free quantification (LFQ) using IMP-apQuant without a match in the run mode.^26^

Cross-linked peptides were identified using MS Annika^27^ (v1.0.18345) within PD v2.5.0.400. The workflow tree consisted of the MS Annika Detector node (MS tolerance 10 ppm, cross-link modification: DSBU +196.085 Da at lysine, and doublet pair selection in the combined mode) followed by the MS Annika Search (full tryptic digest, 5/10 ppm peptide/fragment mass tolerance, max 3 missed cleavages, and carbamidomethyl +57.021 Da at cysteine as static and oxidation +15.995 Da at methionine as the dynamic modification) and completed with the MS Annika Validator (1% FDR cutoff at the cross-link specific match (CSM) and cross-link (XL) level and, separate intra/inter-link FDR is set to false). Relative abundances of the identified cross-linked peptides were determined by LFQ without match between runs using IMP-apQuant.^26^ The search was performed against a database containing*Streptococcus pyogenes*Cas9 and 116 CRAPome proteins.^28^ For FDR control, peptides cross-linked within the same group (as defined previously) were considered correct, and link-connections between peptides of different groups or to peptides from the contaminant database were considered incorrect.

## Results and Discussion

### Column Benchmarking

**Figure 1:**
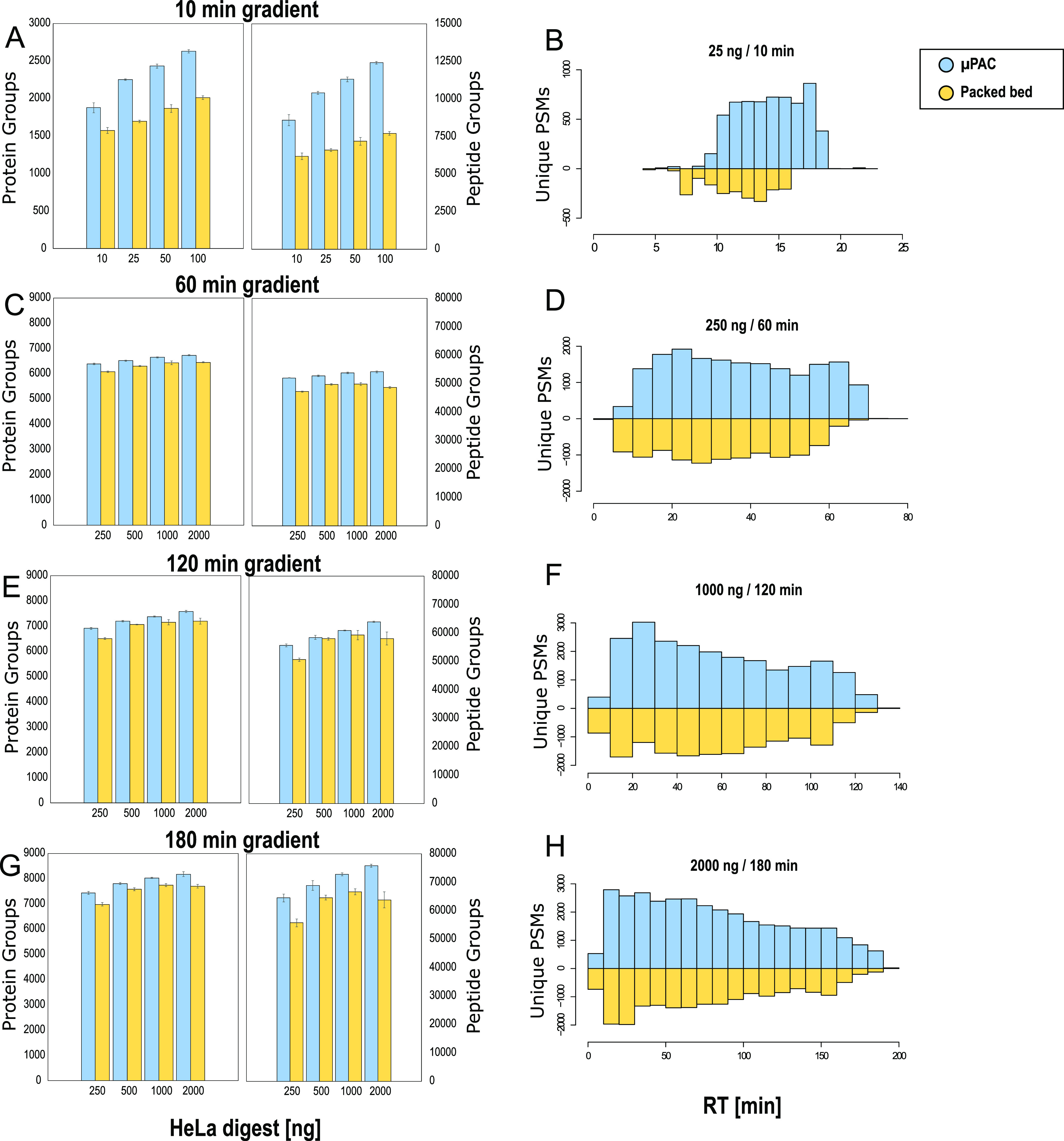
Proteome coverage (protein and peptide group ID’s) obtained for different gradient lengths and sample loads during the extensive benchmarking experiment. Four different methods are tested, and the generation 2 pillar array column (blue) is compared to a packed bed column (yellow). All values represent average values (*n* = 3, injection replicate), with error bars depicting standard deviations. Unique PSMs identified on each column are plotted as a function of elution time to the right. (A,B) 10 min gradient separation, 3 CV FAIMS method, and 10–100 ng of HeLa digest sample’ (C,D) 60 min gradient separation, 3 CV FAIMS method, and 250–2000 ng of HeLa digest sample; (E,F) 120 min gradient separation, 3 CV FAIMS method, and 250–2000 ng of HeLa digest sample; (G,H) 180 min gradient separation, 4 CV FAIMS method, and 250–2000 ng of HeLa digest sample.

After optimization of a confined set of LC and MS parameters (information provided in Supporting Information, Figures S3–S6 and Tables S1–S3), we performed a comprehensive benchmarking experiment to evaluate the column’s applicability for a range of LC gradient settings. The prototype μPAC column was benchmarked against a commercially available packed bed nanoLC column using the conditions listed in Table S4. When applying a short 10 min gradient (Figure 1A), over 2600 proteins could repeatedly be identified from 100 ng of HeLa digest. A significant increase in both peptide and protein group identifications was observed when comparing the micropillar array and the packed bed column (student *t*-test, *p* < 0.001). Even though the processed results did not reveal a significant impact on the chromatographic performance (peak capacity—Figure S7, median FWHM of peptides—Figure S8) and the difference in column void times was found to be minimal (6.5 min for the packed bed column and 8.5 min for the μPAC column), 20–30% more protein groups and 40–60% more peptide groups could be identified when using the pillar array format. When plotting the amount of unique PSMs versus the retention time (Figure 1B), a clear trend is revealed with additional unique identifications toward the end of the gradient. These data suggest that the column morphology has an impact on the elution behavior of hydrophobic peptide species. Consistent with the results reported on the use of superficially porous and large mesopore-size stationary phases, we hypothesize that the use of superficially porous rather than fully porous chromatographic media promotes elution and prevents persistent adsorption of analytes to the chromatographic support material. Additional data that confirm this statement are provided when evaluating sample carryover and analyzing cross-linked peptides on both LC column formats. It must be noted that the use of superficially porous stationary phases brings along some limitations, as they typically have lower loading capacities and show poor retention of hydrophilic peptides. Another consideration concerning these fast gradients for low sample amounts is that these methods are far from optimal when maximum instrument occupation efficiency is pursued, as it takes 35 min to have 10 min of peptide elution.

More efficient MS utilization can be achieved when using longer solvent gradients (75% for 60, 86% for 120, and 90% for 180 min; MS efficiency calculation is provided in Supporting Information). However, protein identification rates observed for short gradients attenuate according to the gradient length. This can be attributed to the fact that the first proteins to be identified from a complex mixture are highly abundant ones that can be picked up relatively easily. Further increases in proteomic depth progressively become more challenging as undiscovered proteins are of ever decreasing abundance. This is clearly illustrated in Figure S10, where the abundance of proteins uniquely discovered by extending the gradient length or the sample load has been compared to those shared with shorter analyses. The relative increase in protein identifications fades with the increasing gradient length, reaching an averaged maximum of close to 8100 protein groups identified out of 2 μg of the HeLa digest sample (Figure 1C,E,G). Again, consistently more features were identified when using the pillar array as compared to the packed bed column. Even though the relative increase in identifications was smaller as compared to the high throughput method (3–6% on the protein group level and 6–19% on the peptide group level), unique hits were again predominantly originating from later-eluting peptide species (Figure 1D,F,G), confirming earlier observations.

### Artifactual Methionine Oxidation of Peptides

**Figure 2:**
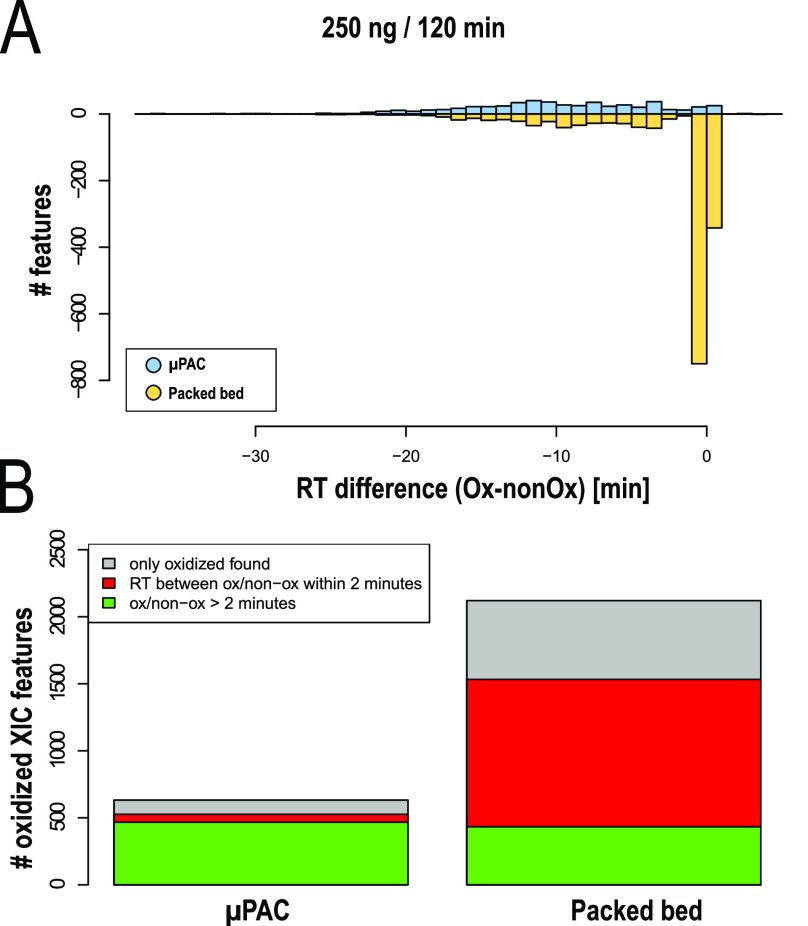
Comparison of peptide oxidation levels between LC column setups. (A) Number of oxidized methionine-containing peptides plotted as a function of the retention time difference between the nonoxidized and the oxidized forms—RT difference (ox—nonOx). Generation 2 pillar array column setup (blue) vs the packed bed column setup (yellow). (B) Total number of oxidized methionine-containing features found in different column setups.

When comparing both column setups, a significantly higher portion of peptides containing oxidized methionine residues was identified with the packed bed column (Figure 2B). Even though methionine oxidation of peptides is often biologically relevant (in vivo modification), for instance, as observed in a range of oxidative stress and age-related disease states, sample handling and analysis can induce artifactual oxidation (in vitro oxidation) and lead to a biased interpretation of biological results. Artifactual oxidation can occur at different stages of a typical bottom-up proteomics workflow, ranging from protein storage and purification to LC separation and ionization.^29^ When oxidized species are present within the sample prior to the reversed phase LC (RPLC) analysis, a retention time difference between the oxidized and nonoxidized forms of the methionine containing peptide is typically observed. The oxidation of a methionine residue to methionine sulfoxide or methionine sulfone reduces hydrophobicity and therefore results, in most cases, in reduced RPLC retention.^30^ LC separation or electrospray ionization-induced oxidation, on the other hand, has much less impact on the peptide retention behavior.^31^ Peptides are not yet present in their oxidized form upon injection onto the LC column and therefore elute much closer to their nonoxidized form. When plotting the amount of oxidized methionine containing peptides as a function of the relative retention time difference with their nonoxidized form (Figure 2A), clear differences are observed between both column setups. Significantly more oxidized features with retention time differences smaller than 2 min are detected when working with the pulled tip emitter column setup (paired *t*-test between two sample groups from 60, 120, and 180 min gradients, *p* = 0.0127). Up to 54% of oxidized species show retention time differences below 2 min, whereas this is only 11% when working with the μPAC column setup.

As both columns had very limited operational history (≤100 runs, only “clean” digest standards) prior to these analyses and samples were always freshly reconstituted from lyophilized HeLa digest pellets, we suggest that the electrical configuration is the most probable source of on column methionine oxidation. Pulled tip emitter column types require upstream high voltage supply, whereas, in contrast, μPAC columns are recommended (or “need”) to be plumbed in such a way that a grounded liquid junction shields any effect of the high voltage that is applied downstream at the emitter (Figure S2). The difference observed in oxidized species is consistent with observations described by Liu et al.,^32^ hypothesizing on-column oxidation by electrochemically formed radicals when a high potential is applied upstream.

### Carryover

**Figure 3:**
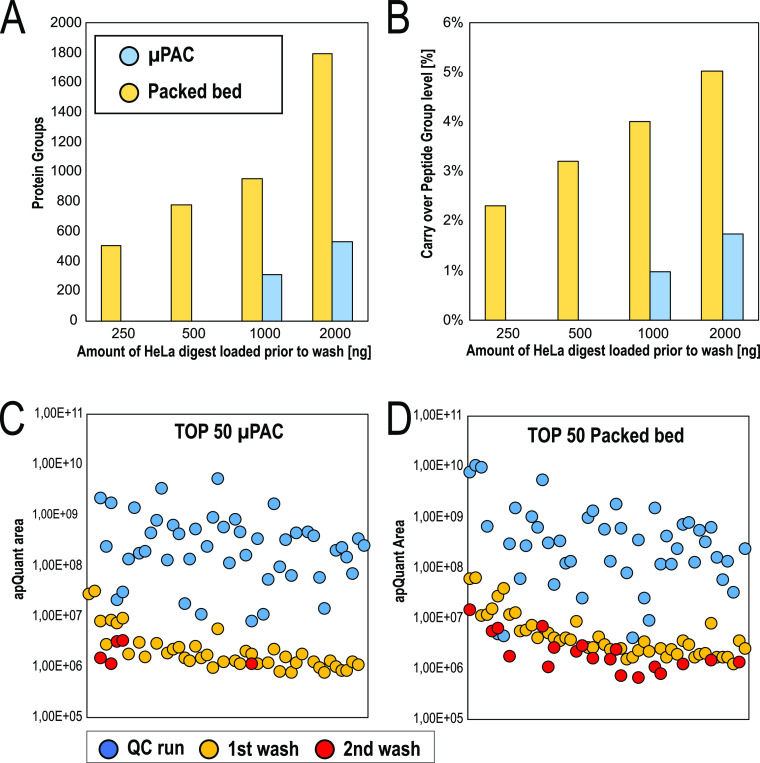
Comparison of sample carryover obtained after increasing sample loads. Blank wash runs immediately after each concentration have been analyzed. Comparison of the generation 2 pillar array (blue) with packed bed column (yellow). (A) Number of protein group ID’s, (B) Relative percentage of carryover on the peptide group level, (C,D) Comparison of apQuant area obtained for top 50 most abundant HeLa peptides; results from the 100 ng QC run are compared with those from the first and the second wash, [(C) = generation 2 pillar array column, (D) = packed bed column].

In many cases, very few or no intermediate washes are performed between runs. It is often assumed that a single blank injection is sufficient to clear persistent sample material without actually acquiring or analyzing MS/MS data. In practice, these assumptions can have a serious impact on results and affect the outcome of a study. The newly introduced Vanquish Neo UHPLC enabled an unbiased investigation of LC column related sample carryover, as after each injection and in parallel to the peptide separation step, the autosampler executes stringent system washing cycles with a high volume of organic solvent to wash the needle outside and the complete injection fluidics path, including the needle inside. To assess LC column-related sample carryover, blank injections were included in the benchmarking series. Figure 3A shows the number of protein groups identified from blank runs for both column setups. Up to a sample load of 1 μg, no protein groups were identified from the blank injections on the μPAC prototype, as there were too few spectra present for FDR assessment in the percolator (200 peptides required).^25^ More data is provided by analyzing the results obtained for consecutive washes (*n* = 2) that have been performed after the 100 ng HeLa QC runs. Using a fixed value validator for FDR assessment, apQuant areas obtained for the top 50 most abundant peptides have been compared (Figure 3C,D). Wash runs performed immediately after the analytical run (1^st^ wash) still show up quite some quantifiable signals for both columns; 43 and 46 out of 50 peptides were quantified on the pillar array and packed bed, respectively. There is, however, a significant difference when analyzing data from the second wash run. 5 and 19 peptides were quantified in the second wash. As mentioned before, when discussing the impact of stationary phase support morphology on peptide elution, we believe this is a result of the intrinsic difference in the interaction surface between both column types. This consistently results in decreased carryover-related identifications on the μPAC column, 3–4 times less on the peptide group level, and 2–3 times less on the protein group level. Additional experiments with packed bed columns that contain superficially porous particles might provide additional insights to support our statements.

### Performance Consistency

Similar to the initial column installation, we implemented a quality control method to assess performance at regular intervals over time. To limit the impact on total acquisition time, a 15 min gradient separation of 100 ng of HeLa digest with a total cycle time of 35 min in between runs was used. Consistent performance was obtained throughout the period of 1 month, which was the time needed to run MS optimization, LC optimization, and actual in-depth benchmarking of a single column. During this period, a slight decrease in protein group IDs (approximately 14%) was observed, going from 3382 to 2979 protein groups with a single column to emitter assembly. A clear effect is, however, observed when the pillar array was replaced by a packed bed column, clearly marked by a sudden drop in identifications (Figure S11). Similar proteome depth was never achieved with the packed bed column, resulting in an average of about 2595 protein groups over a 10-day period of analysis time. To confirm that these observations were linked to the LC column type rather than to the MS performance or to batch effects, a second pillar array column was installed immediately after the packed bed column benchmark. This event is again marked by a distinct increase in identifications (Figure S11). Additional experiments to investigate μPAC column-to-column reproducibility were conducted more than a year after the benchmarking experiments. With a similar setup but now coupled to an Orbitrap Exploris 480 instrument, the performance of 3 prototype μPAC generation 2 columns was compared for 30 min gradient separations. Results have been compiled in the Supporting Information (Figure S12), showing inter column retention time variation below 1% CV and providing clear proof for consistent performance in bottom-up proteomics with a variation on identified protein and peptide groups below 1 and 2% CV, respectively.

### Cross-Linking Experiments

In addition to providing a column performance comparison for standardized HeLa digest samples, we performed a limited set of experiments with cross-linked peptide samples. During the last decade, cross-linking mass spectrometry was established as a potent technique to investigate protein–protein interaction networks as well as in the field of structural proteomics. This technique, including a wide variety of applications, has already been described in several excellent reviews.^33−35^ Briefly, two amino acid residues are covalently connected by application of the cross-linker reagent, followed by proteomic sample preparation, yielding two interconnected peptides for detection by mass spectrometry. Depending on the used linker type, cross-linkers can target amines (lysines), sulfhydryl groups (cysteines), carboxylic acids (glutamic- or aspartic-acid), or they can form radical species reactive to any amino acid. The broad variety of linker types, acquisition techniques, and data analysis algorithms makes it difficult to find an optimal workflow for a specific protein system. To alleviate this issue, we previously developed a synthetic peptide library based on sequences of the Cas9 protein.^23^ The peptides contain exactly one targeted (i.e., lysine) amino acid for cross-linking. They were mixed into groups that were separately cross-linked, followed by quenching and pooling to a single peptide library. In contrast to experiments where the FDR is computationally determined only by applying the known target–decoy approach, this system allows an exact FDR calculation as only interpeptide connections within a group are possible.^36^ Furthermore, the maximal theoretical cross-link number is known (426 unique combinations), which allows for estimating the efficiency of a detection workflow based on the reached identification numbers. Such a synthetic library, in combination with the linker reagent (DSBU), therefore represents an ideal benchmarking tool for the comparison of two different chromatographic setups, as done in this study.

**Figure 4:**
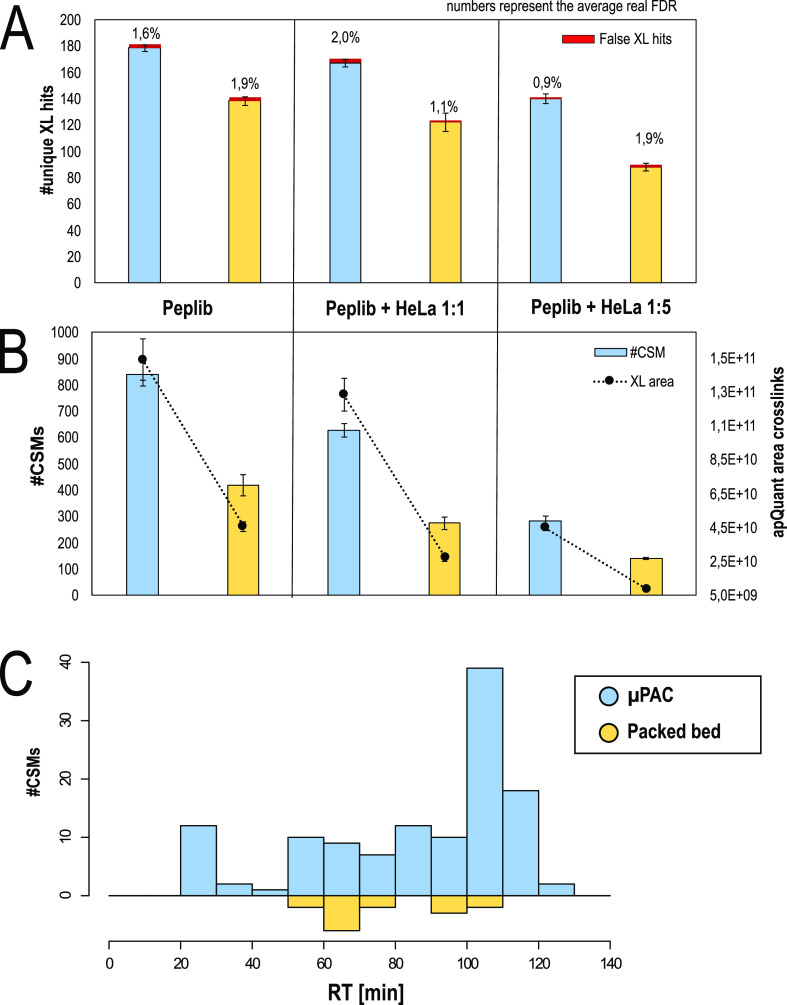
Benchmarking of the generation 2 pillar array column vs the packed bed column using a DSBU cross-linked synthetic peptide library. (A) Number of unique cross-links identified on the 1% estimated FDR level and real FDR printed above. (B) Number of identified cross-linked peptides (CSMs) and its relative abundance based on LFQ. (A,C) All values represent average values (*n* = 3, injection replicate), with error bars depicting standard deviations. (C) Number of cross-linked peptides exclusively identified with either a pillar array or a packed bed chromatographic setup vs the retention time in on the representative replicate, summed to 10 min windows.

The number of identified unique cross-links, as well as the number of cross-link spectrum matches, is reproducibly boosted when using the pillar array column setup compared to the packed bed setup (Figure 4A,B). The number of identifications decreases upon increasing the background of linear peptides present in the sample, which is likely not only a result of increased sample complexity but also of decreased amounts of cross-linked peptides present in spiked samples (i.e., 1 μg of XL-peptides without spiking vs 200 ng of XL peptides + 800 ng of tryptic HeLa peptides in the 1:5 spiked sample). Of note is that the advantage of the pillar array over a packed bed increases in complex sample mixtures. On average, we observed a boost in cross-link IDs of ∼29% without spiking but of 37 and 59% upon 1:1 and 1:5 spiking, respectively. In line with ID numbers, also, the relative abundance of cross-linked peptides is increased in the pillar array setup for all three test samples. The average real FDR rate is close to the expected 1% in all sample types and is independent of the used column, highlighting the quality of the obtained data and a properly working target decoy-based FDR approach using MS Annika. As shown in Figure 4C and in line with the results obtained using different sample loads and gradient lengths (Figure 1B,D,F,H), we observed most of the extra CSM identifications with the μPAC column at high retention times. This could indicate fewer losses of larger species, which are expected to be predominant among cross-linked peptides as two peptides are connected.

## Conclusions

The data compiled in this manuscript provide a transparent perspective on the benefits that next-generation μPAC technology can bring to nanoLC–MS proteomics workflows. By combining this technology with the latest innovations in LC–MS/MS instrumentation, significant improvements in proteome coverage can be obtained with high reproducibility, robust operation, and minimal sample carryover. Improvements in the chromatographic performance were achieved by reducing the pillar diameter and the interpillar distance by a factor of 2, resulting in separation channels being filled with 2.5 μm diameter pillars at an interpillar distance of 1.25 μm. As opposed to packed bed columns with integrated emitter tips, LC column and ESI emitter lifetimes can be detached, providing a potentially more sustainable LC–MS solution without compromising separation performance. After optimization of a confined set of MS and LC parameters, systematically higher proteome coverage could be obtained as compared to pulled-tip packed bed nanoLC columns.

For short gradients (10 min) and limited sample amounts (10–100 ng of cell lysate digest), the impact on proteome coverage was found to be most pronounced, with gains in proteome coverage between 20 and 30% at the protein group level. When extending gradient lengths (60, 120, and 180 min) and injecting sample amounts typically encountered in the analysis of whole cell lysates (250–2000 ng), increases in coverage were less distinct, producing an increase in proteome coverage between 3 and 6% on the protein group level. The highest proteome coverage was obtained with an optimized 180 min gradient separation, where 2 μg of HeLa cell digest was injected, resulting in an average number of identified protein groups of 8100.

A comparison of peptide elution behavior revealed that a larger portion of uniquely identified peptides was acquired at later eluting times, suggesting that the intrinsic difference in surface morphology (superficially porous vs fully porous) produces an alternative distribution of peptides across the solvent gradient. These differences in surface morphology are also thought to be the main contributor to the reduced sample carryover that was observed in the current experiments. Column-related carryover could be reduced by a factor of 2–3 by switching from fully porous packed beds to superficially porous microfabricated column types. In-depth investigation of carryover revealed that at least 2 wash cycles were needed to wash away highly abundant peptides on traditional fully porous silica-based LC columns. Conversely, only a single wash cycle was sufficient when operating μPAC columns, thereby providing better quality data at increased instrument productivity.

Even though benchmarking studies and LC–MS/MS instrument optimization are typically performed with highly validated mammalian protein digest standards, results are often interpreted as deceiving as the experiments are performed under ideal sample loading and composition conditions. To verify the results obtained in the benchmarking study, both column types were subsequently used in the analysis of a DSBU-crosslinked synthetic library. When using the μPAC column, 29 to 59 more unique crosslinked peptides could be identified at an experimentally validated FDR of 1–2%, providing additional proof for the general applicability of the next-generation μPAC technology in a range of nanoLC–MS proteomics workflows.

## Supporting Information Available

MS settings optimization; FAIMS setting selection; LC conditions optimization; representation of the unit cells used to design pillar array column generations; overview of the fluidic and electrical configurations; impact of MS settings on the identification rate, MS scanning speed, and MS2 identification rate; impact of FAIMS pro compensation voltage settings on proteome coverage; impact of the flow rate on the identification rate; LC/MS settings used during MS method optimization, FAIMS optimization, LC flow rate optimization, and the final benchmarking experiment; FWHM-based peak capacity for different column types; violin plots; venn diagrams; comparison of the apQuant area; evolution of column performance; and column reproducibility evaluation (PDF)
